# Value of variation index of inferior vena cava diameter in predicting fluid responsiveness in patients with circulatory shock receiving mechanical ventilation: a systematic review and meta-analysis

**DOI:** 10.1186/s13054-018-2063-4

**Published:** 2018-08-21

**Authors:** Haijun Huang, Qinkang Shen, Yafen Liu, Hua Xu, Yixin Fang

**Affiliations:** Emergency Department, The First Affiliated Hospital of Zhejiang Chinese Medical University, Zhejiang, 310018 Hangzhou China

**Keywords:** Fluid responsiveness, Inferior vena cava diameter, Mechanical ventilation, Meta-analysis

## Abstract

**Background:**

Respiratory variations in the inferior vena cava diameter (ΔIVCD) have been studied extensively with respect to their value in predicting fluid responsiveness, but the results are conflicting. The aim of this meta-analysis was to explore the value of ΔIVCD for predicting fluid responsiveness in patients with circulatory shock receiving mechanical ventilation.

**Methods:**

PubMed, Embase, and the Cochrane Central Register of Controlled Trials were searched up to June 2017. The diagnostic OR (DOR), sensitivity, and specificity were calculated. The summary ROC curve was estimated, and the area under the ROC curve (AUROC) was calculated.

**Results:**

Overall, 603 patients were included in this review, 324 (53.7%) of whom were fluid-responsive. The cutoff values of ΔIVCD varied across studies, ranging from 8% to 21%. Heterogeneity between studies was assessed with an overall *Q* = 0.069, *I*^2^ = 0%, and *P* = 0.483. The pooled sensitivity and specificity for the overall population were 0.69 (95% CI, 0.51–0.83) and 0.80 (95% CI, 0.66–0.89), respectively. The DOR was 9.28 (95% CI, 2.33–36.98). AUROCs were reported in five studies. Overall, the pooled AUROC was 0.82 (95% CI, 0.79–0.85).

**Conclusions:**

The findings of this study suggest that the ΔIVCD performed moderately well in predicting fluid responsiveness in patients with circulatory shock receiving mechanical ventilation.

**Electronic supplementary material:**

The online version of this article (10.1186/s13054-018-2063-4) contains supplementary material, which is available to authorized users.

## Background

Fluid resuscitation remains the cornerstone of treatment for patients with acute circulatory failure. Inappropriate administration of fluids has deleterious effects, including volume overload, systemic and pulmonary edema, and limitation of oxygen diffusion to tissues, thereby leading to increased tissue hypoxia [1–3]. Therefore, it is important to obtain reliable information about fluid responsiveness in patients with circulatory failure in the intensive care unit. However, clinicians often have inaccurate, nonspecific information to guide treatment.

Previous studies have shown that some parameters may be related to volume status. The traditional static parameters, such as intrathoracic blood volume index, pulmonary wedge pressure, pulse pressure variation, and central venous pressure, have been proved not to be related to a patient’s volume status [1, 2]. Hemodynamic parameters, such as pleth variability index and stroke volume variation, may better predict fluid responsiveness. However, the measurement of these parameters requires invasive procedures and special monitoring equipment, limiting their clinical application [3].

In recent years, ultrasound has been considered as a tool to help guide fluid resuscitation [4]. Respiratory variation in the inferior vena cava diameter (ΔIVCD) measured by ultrasonography has been identified as a predictor of dry weight in patients undergoing hemodialysis [5, 6]. ΔIVCD has been identified as a predictor of fluid responsiveness in spontaneously breathing patients in different clinical settings [7, 8]. The ΔIVCD under mechanical ventilation has also been proven as a reliable noninvasive indicator of fluid responsiveness in patients with sepsis [9, 10].

The present systematic review and meta-analysis was conducted to assess the diagnostic accuracy of ΔIVCD for predicting fluid responsiveness in patients with circulatory shock receiving mechanical ventilation. In this systematic review and meta-analysis, the test characteristics of ΔIVCD are summarized as a predictor of fluid responsiveness in patients with circulatory shock receiving mechanical ventilation to elucidate further their diagnostic performance and to provide information for detecting fluid responders.

## Methods

This meta-analysis was conducted according to the Preferred Reporting Items for Systematic Reviews and Meta-Analyses guidelines [11].

### Search strategy

Relevant studies up to June 2017 were searched in the PubMed, Embase, and Cochrane Library databases with the following terms and their combination: “fluid therapy,” AND “inferior vena cava,” AND “mechanical ventilation,” AND (“acute circulatory failure” OR “shock”). All scanned abstracts, studies, and citations were reviewed. Moreover, references of the retrieved manuscripts were also manually cross-searched for further relevant publications.

### Selection criteria

All the studies on the requirements for ΔIVCD were evaluated by two independent authors, and any disagreement was resolved by group discussion until a consensus was reached. The inclusion criteria were as follows: (1) studies on patients with shock receiving mechanical ventilation; (2) studies with a reference gold standard for diagnosing fluid responsiveness; (3) studies published in any language; and (4) studies providing sufficient data for constructing two-by-two tables, including true-positive (TP), false-positive (FP), true-negative (TN), and false-negative (FN). The exclusion criteria were as follows: (1) studies that used the same population or overlapping databases and (2) studies on animal models.

### Data extraction and quality assessment

All the available data were extracted from each study by two investigators independently according to the aforementioned inclusion criteria, and any differences were resolved by discussion with a third investigator. The following data were collected from each study: (1) basic characteristics of studies, including name of the first author, publication year, country where the research was performed, sex, mean age, number of patients, tidal volume, index test, reference standard measurement, reference standard threshold, and reference standard device; and (2) diagnostic performance, including cutoff value, sensitivity, specificity, area under the ROC curve (AUROC), TP, FP, FN, and TN. The quality of included studies was scored independently by two reviewers using the revised Quality Assessment of Diagnostic Accuracy Studies (QUADAS-2) criteria [12]. The quality of studies was assessed using RevMan (version 5.3, 2014; The Nordic Cochrane Centre, The Cochrane Collaboration, Copenhagen, Denmark), with four key domains: patient selection, index test, reference standard, and flow and timing. Seven questions were used to evaluate the quality of included studies. Each question was answered with “yes,” “no,” or “unclear.” An answer of “yes” meant that the risk of bias could be judged as low, whereas an answer of “no” or “unclear” meant that the risk of bias could be judged as high. In the case of conflict, a third reviewer was consulted, and disagreement was settled through multilateral discussion.

### Statistical analysis

All analyses were performed using Stata 14.0 software (StataCorp, College Station, TX, USA). The bivariate meta-analysis model was used to summarize sensitivity, specificity, positive likelihood ratio, negative likelihood ratio, and diagnostic OR (DOR) [13, 14]. The sensitivity and specificity of each included study were used to plot the summary ROC (SROC) curves and calculate the area under the SROC curve (AUC). The AUC could be statistically interpreted as the probability of distinguishing patients from normal control subjects correctly. The between-study heterogeneity was evaluated using *Q* test and *I*^2^ statistics. A *P* value less than 0.10 for the *Q* test or *I*^2^ value ≥ 50% indicated substantial heterogeneity, and then the random-effects model was applied. Confirming the stability of the present study, the sensitivity analysis and outliner exclusion were also performed. Because publication bias is a concern for meta-analyses, the Deeks’ funnel plot asymmetry test was used, with *P* < 0.10 indicating statistical significance [15].

## Results

### Characteristics of the studies

**Fig. 1:**
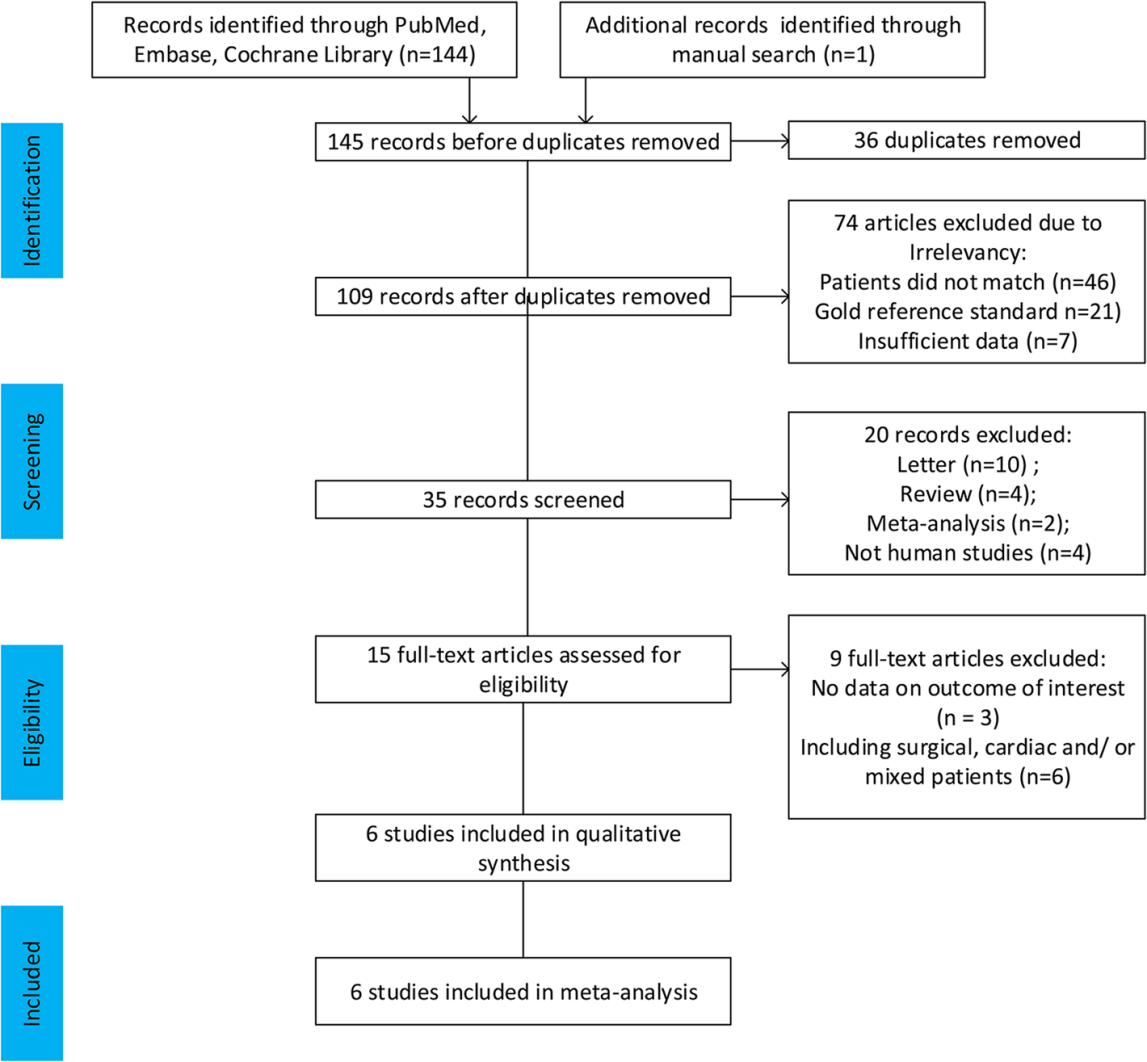
Flow diagram of identification of studies

**Fig. 2:**
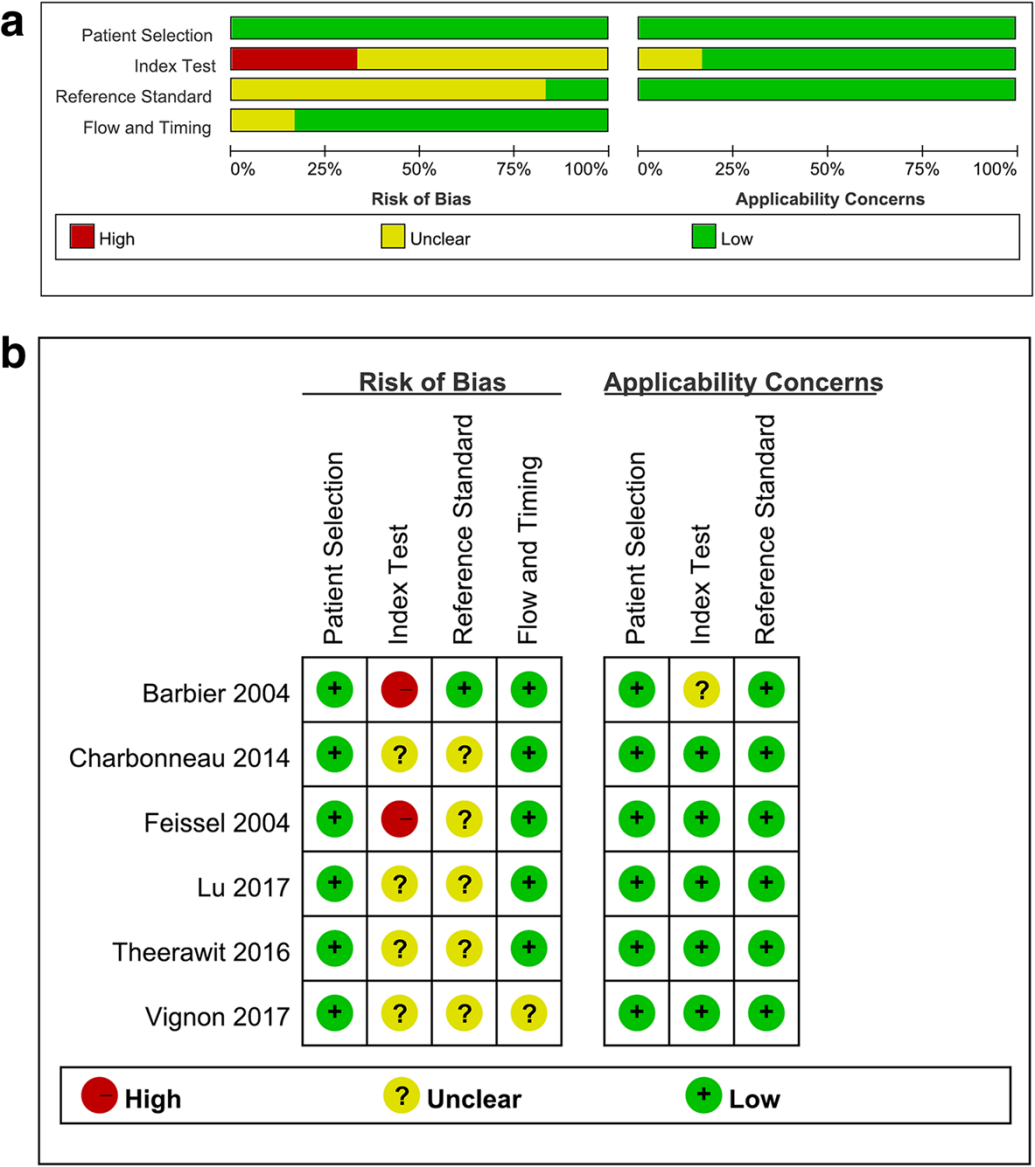
Risk of bias and applicability concerns for the studies included in the meta-analysis. **a** Risk-of-bias graph. **b** Risk-of-bias summary

This meta-analysis yielded 145 primary studies after the initial independent review, comprising 144 published studies identified through electronic database searches and 1 published study identified through a manual search. Figure 1 shows the study selection process. A total of 36 studies were initially excluded owing to duplicate records; 94 studies were excluded owing to the source not being related to the research topic or being a letter, review, or meta-analysis; and 9 studies were excluded because they included surgical, cardiac, or mixed patients or did not present usable data. Finally, six studies [9, 10, 16–19] fulfilled all the inclusion criteria and were considered for analysis. The main characteristics of the eligible studies are shown in Table 1. The quality of the included studies was assessed using QUADAS-2, as shown in Fig. 2.

**Table 1:** Characteristics of studies included in this meta-analysis

First author/year of publication	Country	Sex (M/F)	Age in y (mean ± SD)	Cases	Tidal volume (ml/kg)	Index test	Reference standard measurement	Reference standard threshold	Reference standard device
Barbier 2004 [14]	France	15/5	63 ± 15	20	8.5	(*D*_max_ – *D*_min_)/*D*_min_	CI	> 15%	TTE
Feissel 2004 [15]	United States	22/17	65 ± 15	39	8–10	(*D*_max_ – *D*_min_)/[(*D*_max_ + *D*_min_)/2]	CO	> 15%	TTE
Charbonneau 2014 [16]	France	26/18	58.5	44	8–10	(*D*_max_ – *D*_min_)/*D*_min_	CI	> 15%	TTE
Theerawit 2016 [17]	Thailand	11/18	62.6 ± 15.9	29	8	(*D*_max_ – *D*_min_)/[(*D*_max_ + *D*_min_)/2]	CO	> 15%	PCA
Lu 2017 [18]	China	33/16	R: 55.7 ± 12.6 N: 55 ± 12.8	49	8–10	(*D*_max_ – *D*_min_)/*D*_min_	CI	≥ 10%	TTE
Vignon 2017 [19]	France	379/161	65 ± 13	540	8	(*D*_max_ – *D*_min_)/*D*_min_	SV	> 10%	TTE

*Abbreviations: CI* Cardiac index, *CO* Cardiac output, *D*_max_ and *D*_min_ Maximum (inspiration phase) and minimum (expiration phase) diameter of inferior vena cava over a complete respiratory cycle, respectively, *N* Nonresponder, *PCA* Pulse contour analysis, *R* Responder, *SV* Stroke volume, *TTE* Transthoracic echocardiogram

### Quantitative synthesis

**Fig. 3:**
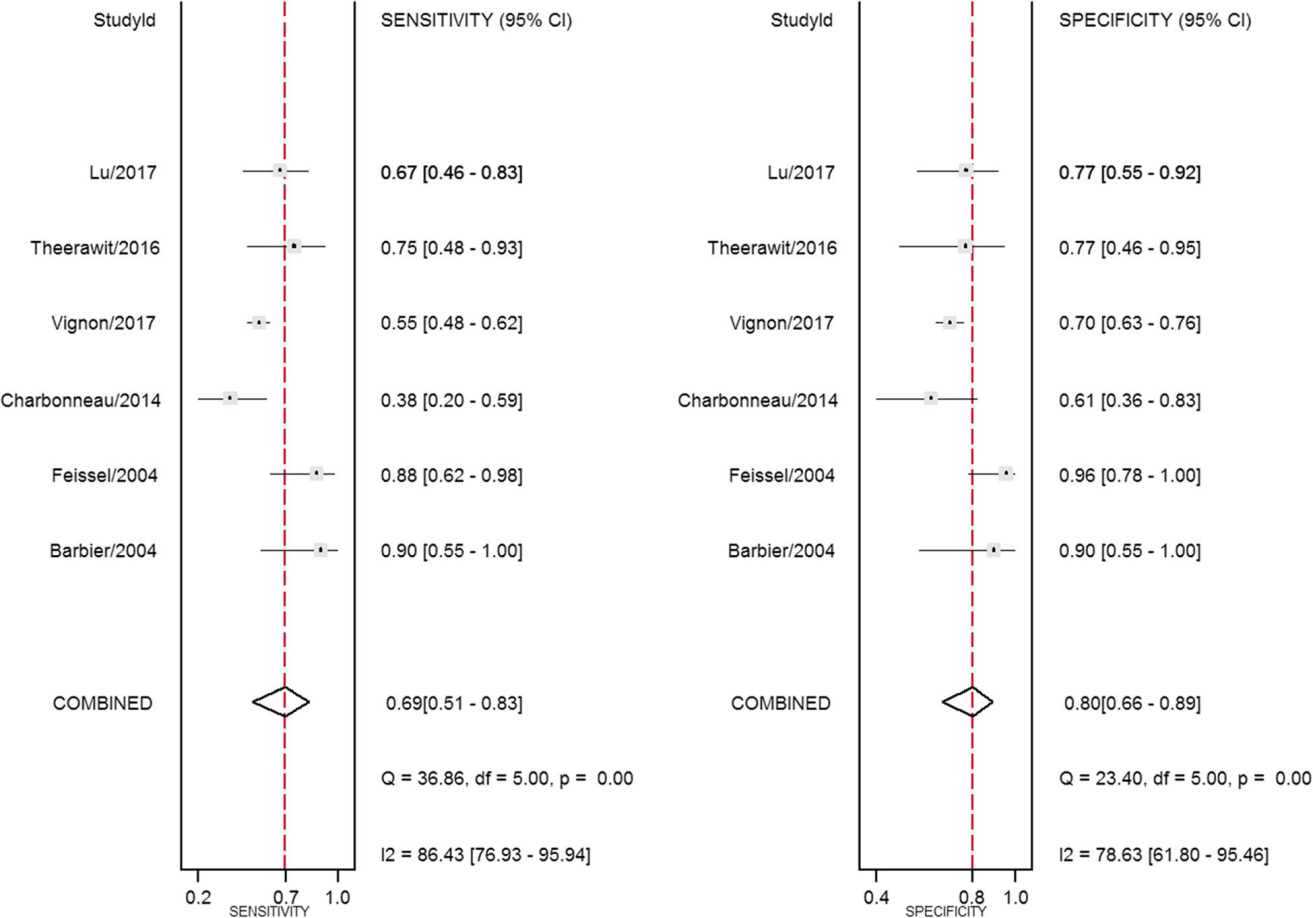
Forest plots of the pooled sensitivity and specificity analysis. Each *solid square* represents an individual study. Error bars represent 95% CI. *Diamonds* indicate the pooled sensitivity and specificity for all of the studies

**Fig. 4:**
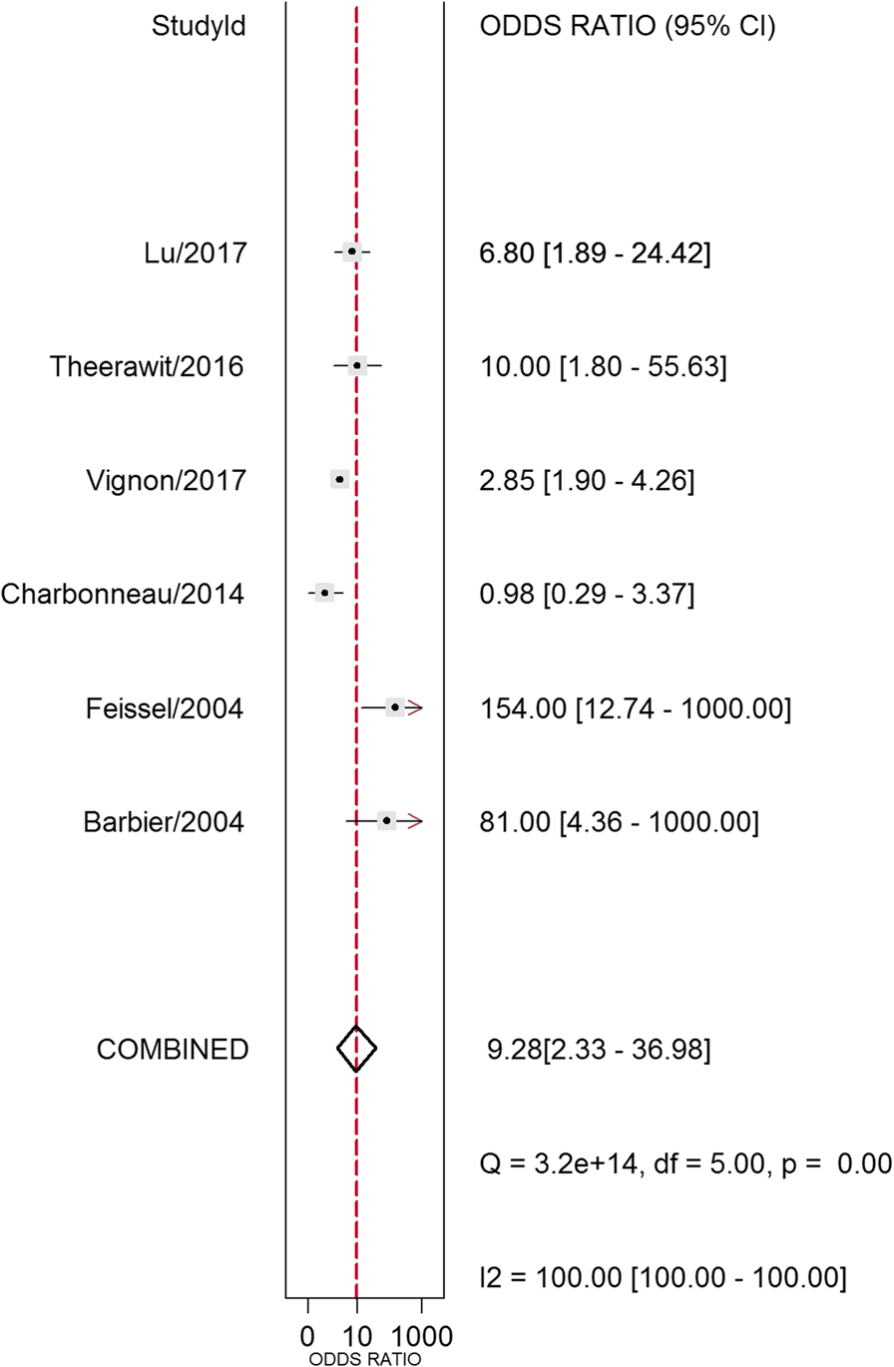
Forest plots of the pooled diagnostic odds ratio. Each *solid square* represents an individual study. Error bars represent 95% CI. *Diamond* indicates the pooled diagnostic OR for all of the studies

**Fig. 5:**
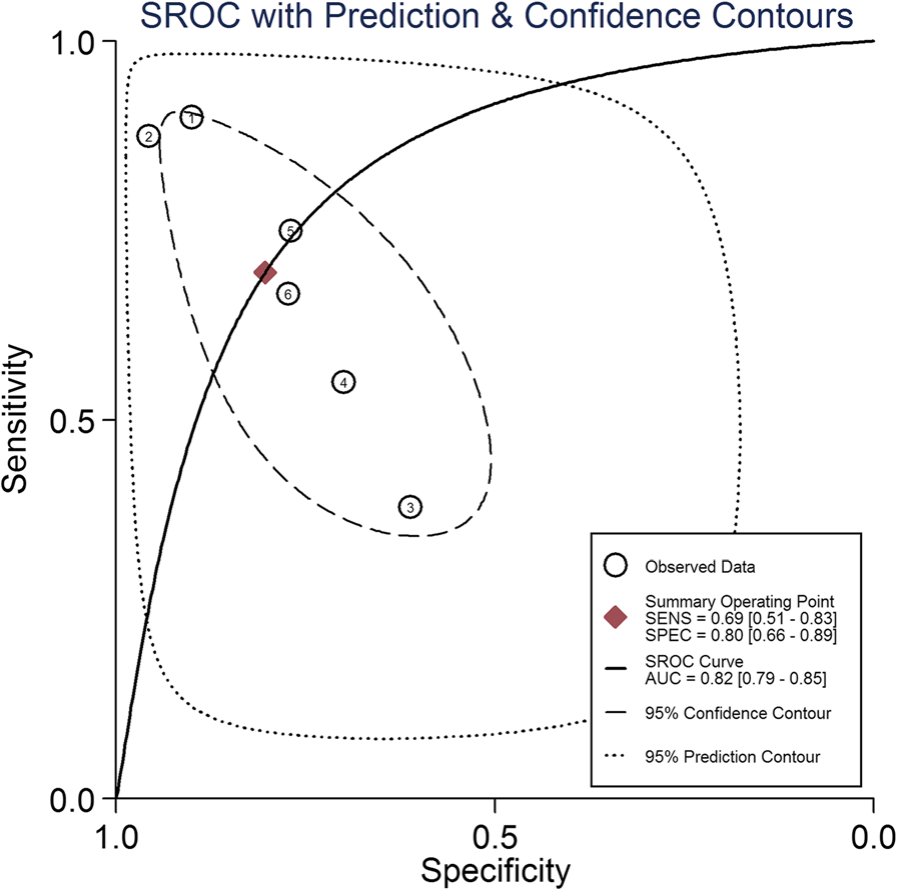
Summary ROC (SROC) curve of respiratory variations of inferior vena cava diameter for predicting fluid responsiveness. Each *circle* represents individual study estimates. The *diamond* is the summary point representing the average sensitivity and specificity estimates. The ellipses around this summary point are the 95% confidence region (*dashed line*) and the 95% prediction region (*dotted line*). The cutoff values of included studies are as follows: (*1*) Barbier 2004 [9], 18%; (*2*) Feissel 2004 [10], 12%; (*3*) Charbonneau 2014 [16], 21%; (*4*) Vignon 2017 [19], 8%; (*5*) Theerawit 2016 [17], 10%; and (*6*) Lu 2017 [18], 20.5%

Study data and individual diagnostic estimates are summarized in Table 2. Overall, 603 patients were included in this review, 324 (53.7%) of whom were fluid-responsive. The cutoff values of ΔIVCD varied across studies, ranging from 8% to 21%. The AUROC of individual studies ranged from 0.43 to 0.91. Heterogeneity between studies was assessed with an overall *Q* = 0.069, *I*^2^ = 0%, and *P* = 0.483. The pooled sensitivity and specificity for the overall population were 0.69 (95% CI, 0.51–0.83) and 0.80 (95% CI, 0.66–0.89), respectively (Fig. 3). The DOR was 9.28 (95% CI, 2.33–36.98) (Fig. 4). AUROCs were reported in five studies [9, 16–19]. In four studies [9, 17–19], the AUROC was > 0.5, and in the remaining study [16], ΔIVCD had no diagnostic value. Overall, the pooled AUROC was 0.82 (95% CI, 0.79–0.85) (Fig. 5).

**Table 2:** Outcomes of studies included in this meta-analysis

First author/year of publication	Sample size	Cutoff (%)	Subject numbers could be calculated				Sensitivity (%)	Specificity (%)	AUROC (95% CI)
			TP	FP	FN	TN			
Barbier 2004 [9]	20	18	9	1	1	9	90	90	0.91 (0.84–0.98)
Feissel 2004 [10]	39	12	14	1	2	22	88	96	NA
Charbonneau 2014 [16]	44	21	10	7	16	11	38	61	0.43 (0.25–0.61)
Theerawit 2016 [17]	29	10	12	3	4	10	75	77	0.67 (0.48–0.89)
Lu 2017 [18]	49	20.5	18	5	9	17	67	77	0.81 (0.67–0.94)
Vignon 2017 [19]	422	8	126	58	103	135	55	70	0.635

*Abbreviations: AUROC* Area under the ROC curve, *FN* False-negative, *FP* False-positive, *NA* Not available, *TN* True-negative, *TP* True-positive

### Sensitivity analyses

Goodness-of-fit and bivariate normality analyses (Additional file 1: Figure S1a and b) showed that the bivariate model was robust for calculating the pooled estimates. Influence analysis and outlier detection (Additional file 1: Figure S1c and d) did not reveal outlier studies, suggesting the robustness of the present meta-analysis.

### Publication bias

The publication bias of the studies was assessed using the Deeks’ funnel plot asymmetry test. The slope coefficient of the six studies was associated with a *P* value of 0.19 (Additional file 2: Figure S2). The aforementioned results indicated symmetrical data and no significant publication bias.

## Discussion

In this study, the diagnostic accuracy of ΔIVCD in predicting fluid responsiveness in patients with circulatory shock receiving mechanical ventilation was evaluated in a systematic review and meta-analysis. The results confirmed that, overall, ΔIVCD performed moderately well in predicting fluid responsiveness in patients with circulatory shock receiving mechanical ventilation, with a pooled AUROC of 0.82 (95% CI, 0.79–0.85). A positive IVC ultrasound was moderately predictive of fluid responsiveness, with a pooled specificity of 0.80 (95% CI, 0.66–0.89). A negative IVC ultrasound, however, could not be used to rule out fluid responsiveness, with a pooled sensitivity of 0.69 (95% CI, 0.51–0.83). Some included studies showed that the measurement of IVCD variation was judged by eye, without overlaying the ventilator waveform, and therefore the sensitivity was low [17]. However, ΔIVCD could be assessed by eye, overlaying the ventilator waveform, and the cutoff of ΔIVCD was unclear. Further diagnostic studies are warranted to obtain the appropriate cutoff value and validate the pooled results. IVCD is a noninvasive, easily obtained measure that may be used to predict fluid responsiveness in multiple patient settings. These findings are clinically relevant because point-of-care ultrasonography is becoming increasingly popular, and ΔIVCD values can be obtained immediately in the emergency or critical care setting.

Authors of two systematic reviews and meta-analyses [20, 21] have investigated the diagnostic performance of ΔIVCD in predicting fluid responsiveness. Recently, Long et al. [20] conducted a comprehensive systematic review and meta-analysis on the value of ΔIVCD in predicting fluid responsiveness. Compared with their study, the present study was focused only on patients with shock receiving mechanical ventilation, whereas Long et al. analyzed a variety of patients with sepsis, including neurosurgery, cardiac, and subarachnoid hemorrhage patients. Additionally, several published studies [18, 19] were not included in that meta-analysis. More eligible studies [16–19] were identified, and in contrast with a recent meta-analysis on the value of ΔIVCD in predicting fluid responsiveness reported by Zhang et al. [21], we performed a detailed analysis in the present study. Zhang et al. analyzed only two of six studies [9, 10] included in our meta-analysis. Moreover, the results of the present meta-analysis were not exactly the same as those of a previous study in terms of the main outcomes assessed, such as sensitivity, DOR, and so forth.

The present systematic review and meta-analysis had some limitations. First, this analysis included only six studies with a relatively small sample size, among which the study performed by Vignon et al. [19] was the largest study with more than 60% of the total sample size. Therefore, the power and precision of the results were limited. Second, the quality assessment showed a high risk of bias in the index test. Two studies were at high risk owing to insufficient information to judge whether their test results were interpreted blind. This bias might have restricted the interpretation of the true diagnostic efficacy of ΔIVCD in predicting fluid responsiveness. Third, because more detailed individual patient data were not available, a more comprehensive analysis of diagnostic effect could not be conducted. Fourth, the relatively small sample size in the included studies led to strong diagnostic factors that might not be significant. Finally, the limitations of the huge variation in IVCD for predicting fluid responsiveness may be explained through closer examination of different formulas, different cutoffs, and the included study characteristics.

## Conclusions

This was the first meta-analysis to evaluate the diagnostic accuracy of ΔIVCD in predicting fluid responsiveness in patients with circulatory shock receiving mechanical ventilation. The results suggest ΔIVCD performed moderately well in predicting fluid responsiveness. Further studies with a larger dataset and well-designed models are required to confirm the diagnostic accuracy and utility of IVCD in predicting fluid responsiveness in patients with circulatory shock receiving mechanical ventilation.

## Additional files

Additional file 1: **Figure S1.** Graphs for sensitivity analyses. **a** Goodness of fit. **b** Bivariate normality. **c** Influence analysis. **d** Outlier detection. (TIF 610 kb)

Additional file 2: **Figure S2.** Deeks’ funnel plot with regression line. (TIF 252 kb)

## Abbreviations

AUC: Area under the summary ROC curve
AUROC: Area under the ROC curve
DOR: Diagnostic OR
FN: False-negative
FP: False-positive
IVCD: Inferior vena cava diameter
ΔIVCD: Respiratory variations in the inferior vena cava diameter
QUADAS-2: Quality Assessment of Diagnostic Accuracy Studies
SROC: Summary ROC
TN: True-negative
TP: True-positive
