# Supplementary figures and images for: Value of variation index of inferior vena cava diameter in predicting fluid responsiveness in patients with circulatory shock receiving mechanical ventilation: a systematic review and meta-analysis
Source: Crit Care. 2018 Aug 21;22:204. doi: 10.1186/s13054-018-2063-4 (PMC6102872; doi:10.1186/s13054-018-2063-4)

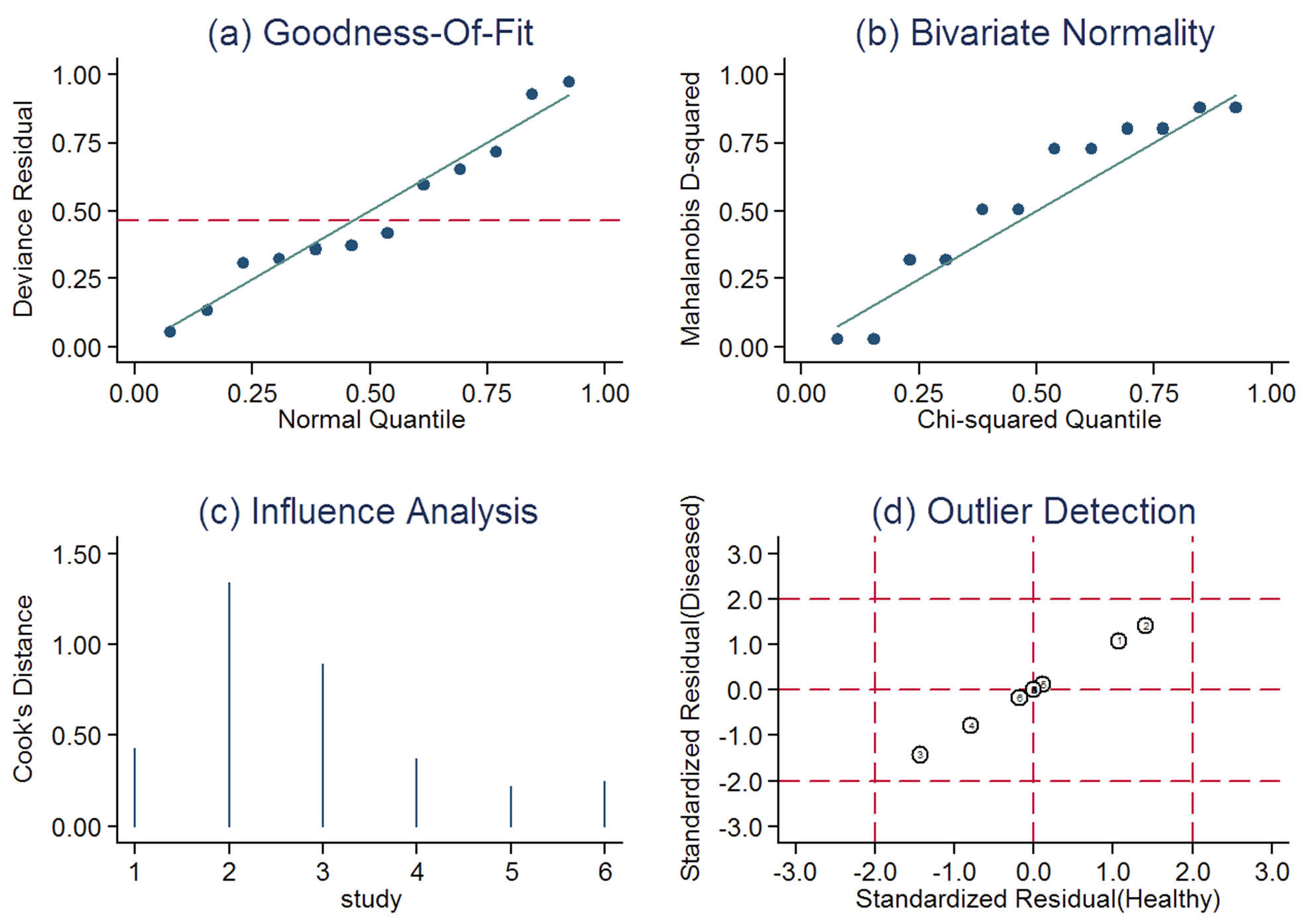

Supplement: Supplementary file 1 — Figure S1. Graphs for sensitivity analyses. a Goodness of fit. b Bivariate normality. c Influence analysis. d Outlier detection. (TIF 610 kb) [file 13054_2018_2063_MOESM1_ESM.tif]

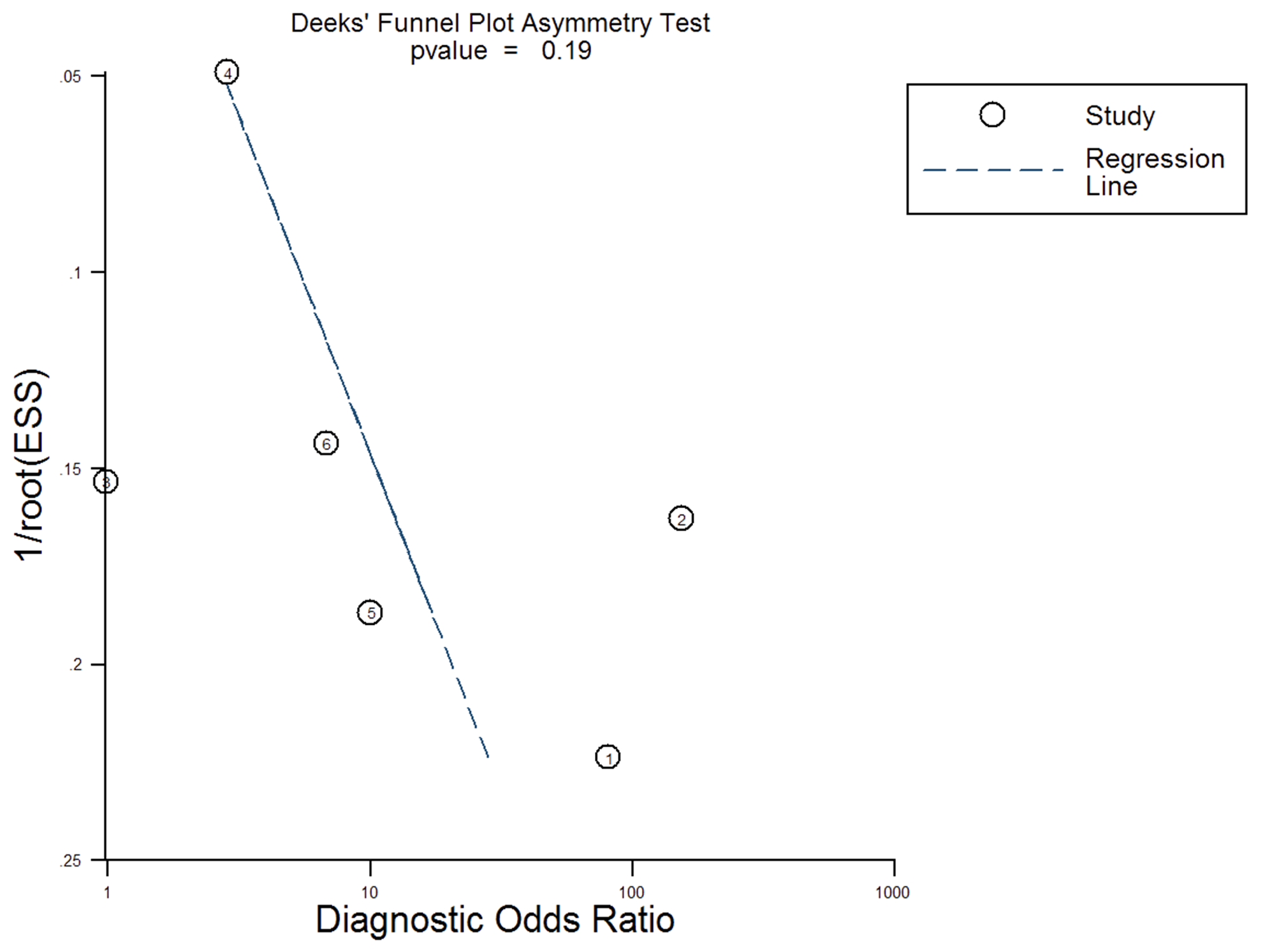

Supplement: Supplementary file 2 — Figure S2. Deeks’ funnel plot with regression line. (TIF 252 kb) [file 13054_2018_2063_MOESM2_ESM.tif]
